# Supplementary material for: Mechanisms governing the pioneering and redistribution capabilities of the non-classical pioneer PU.1
Source: Nat Commun. 2020 Jan 21;11:402. doi: 10.1038/s41467-019-13960-2 (PMC6972792; doi:10.1038/s41467-019-13960-2)
Supplement: Supplementary file 7 — Source data [file 41467_2019_13960_MOESM7_ESM.zip › Source_Data/FigureS2/FigureS2B_MotifScanOutput/KmeansCluster13/homerResults/motif31.similar.html]

motif31

## Information for motif31

C
T
G
A
A
G
T
C
C
G
T
A
A
C
T
G
A
G
T
C
C
G
A
T
A
C
T
G
  
Reverse Opposite:  

G
T
A
C
C
G
T
A
A
C
T
G
G
T
A
C
A
C
G
T
A
C
T
G
G
A
C
T
  

|  |  |
| --- | --- |
| p-value: | 1e-18 |
| log p-value: | -4.251e+01 |
| Information Content per bp: | 1.857 |
| Number of Target Sequences with motif | 672.0 |
| Percentage of Target Sequences with motif | 39.72% |
| Number of Background Sequences with motif | 13249.4 |
| Percentage of Background Sequences with motif | 29.38% |
| Average Position of motif in Targets | 102.7 +/- 58.3bp |
| Average Position of motif in Background | 100.5 +/- 65.5bp |
| Strand Bias (log2 ratio + to - strand density) | 0.0 |
| Multiplicity (# of sites on avg that occur together) | 1.25 |
| Motif File: | file (matrix) reverse opposite |

### Similar de novo motifs found

|  |  |  |  |  |  |  |  |
| --- | --- | --- | --- | --- | --- | --- | --- |
| Rank | Match Score | Redundant Motif | P-value | log P-value | % of Targets | % of Background | Motif file |
| 1 | 0.849 | G T A C G C T A A C T G G A T C G C A T A C T G A C G T C G A T C A T G T A C G T G A C | 1e-15 | -35.382615 | 11.88% | 6.42% | motif file (matrix) |
